# *syn*-Elimination of glutamylated threonine in lanthipeptide biosynthesis†

**DOI:** 10.1039/d2cc06345j

**Published:** 2022-12-14

**Authors:** Raymond Sarksian, Lingyang Zhu, Wilfred A. van der Donk

**Affiliations:** a Department of Chemistry and Howard Hughes Medical Institute, University of Illinois at Urbana-Champaign Urbana IL 61822 USA vddonk@illinois.edu +1 217 244 5360; b School of Chemical Sciences NMR Laboratory, University of Illinois at Urbana-Champaign Urbana IL 61822 USA; c Carl R. Woese Institute for Genomic Biology, University of Illinois at Urbana-Champaign Urbana IL 61822 USA

## Abstract

Methyllanthionine (MeLan) containing macrocycles are key structural features of lanthipeptides. They are formed typically by *anti*-elimination of l-Thr residues followed by cyclization of l-Cys residues onto the (*Z*)-dehydrobutyrine (Dhb) intermediates. In this report we demonstrate that the biosynthesis of lanthipeptides containing the d-*allo*-l-MeLan macrocycle such as the morphogenetic lanthipeptide SapT proceeds through (*E*)-Dhb intermediates formed by net *syn*-elimination of l-Thr.

## Abstract 2

Formation of (*E*)-dehydrobutyrine during lanthipeptide biosynthesis.

## Graphical abstract

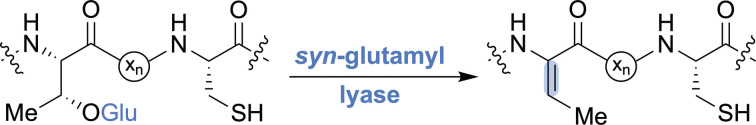

Lanthipeptides are peptidic natural products defined by the presence of lanthionine (Lan) and/or methyllanthionine (MeLan) residues and represent a large class of ribosomally synthesized and post-translationally modified peptides (RiPPs).^1^ One of the most common posttranslational modifications (PTMs) in RiPPs is macrocyclization.^2–7^ Macrocyclic peptides often increase stability towards proteolytic degradation and display more potent bioactivities compared to their linear counterparts.^8^

Macrocyclization for lanthipeptides occurs by thiol-Michael addition of l-Cys to electrophilic dehydroalanine (Dha)/dehydrobutyrine (Dhb) intermediates that are formed by net dehydration of Ser/Thr residues.^1,5,9^ In class I lanthipeptide biosynthesis the dehydration reaction is catalyzed by a LanB enzyme that first activates the side chain of Ser/Thr residues through a transesterification reaction using Glu-tRNA^Glu^ (Fig. 1A).^10–13^ Glutamate elimination by the LanB enzyme in a separate active site then generates Dha/Dhb residues (Fig. 1A). Split LanB enzymes have been reported where the glutamyl transferase and glutamyl lyase (GL) domains are separate polypeptides.^14–18^ The cyclization process for lanthipeptides can generate up to two Lan and four MeLan diastereomers.^1,18,19^ The cyclization reaction is catalyzed by a LanC cyclase for class I lanthipeptides (Fig. 1A).^1,20^ The (*2S*,*6R*)- and (*2R*,*6R*)-Lan diastereomers, hereafter referred to as dl- and ll-Lan, have been detected in lanthipeptides, and three out of four possible MeLan stereoisomers have been observed, (*2S*,*3S*,*6R*)-, (*2R*,*3R*,*6R*)-, and (*2S*,*3R*,*6R*)-MeLan, hereafter denoted as dl-, ll-, and d-*allo*-l-MeLan (Fig. 1B).^18,19,21–25^

**Fig. 1:**
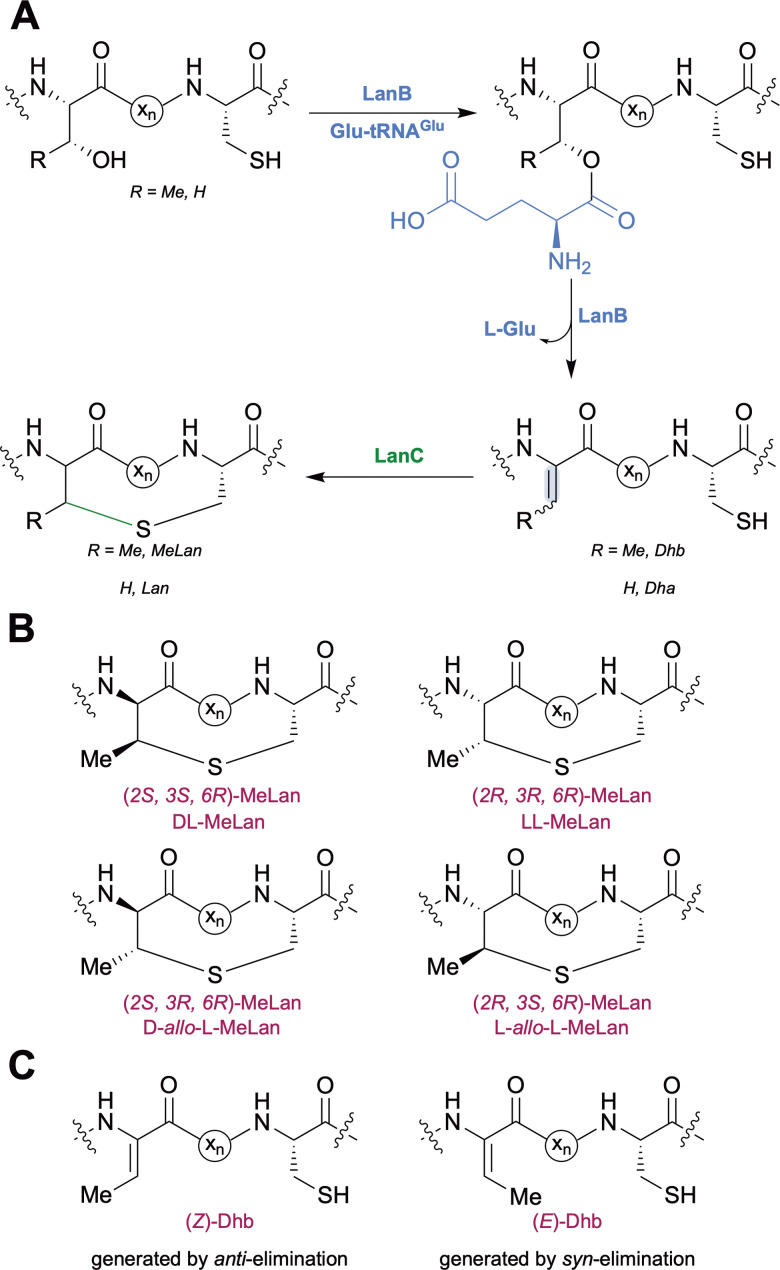
(A) Class I lanthipeptide biosynthesis. (B) Four MeLan stereoisomers. (C) MeLan residues can be generated from either (*Z*)- or (*E*)-Dhb intermediates.

Dehydration of l-Thr residues yields (*Z*)-Dhb in all characterized lanthipeptides (Fig. 1C). dl- and ll-MeLan diastereomers are believed to form by *anti*-addition of l-Cys across (*Z*)-Dhb intermediates; addition across the *Si* face results in dl-MeLan, whereas addition across the *Re* face gives ll-MeLan.^1,21,23^dl-MeLan has been historically observed for most lanthipeptides including nisin,^26^ whereas ll-MeLan has been detected only in the past decade, particularly in substrate-controlled cyclizations.^23,27^d-*allo*-l-MeLan macrocycles were recently discovered in the morphogenetic lanthipeptide SapT (Fig. 2A).^18^d-*allo*-l-MeLan could be formed through *anti*-elimination of l-Thr residues to yield a (*Z*)-Dhb intermediate followed by *syn*-addition of l-Cys across the *Re* face of the (*Z*)-Dhb intermediate.^18^ Alternatively, d-*allo*-l-MeLan can be generated through net *syn*-elimination of l-Thr residues to generate an (*E*)-Dhb intermediate followed by *anti*-addition of l-Cys across the *Si* face of the (*E*)-Dhb intermediate.^18^ A lanthipeptide cyclase has yet to be characterized that catalyzes *syn*-addition and (*E*)-Dhb residues have yet to be observed for a RiPP. In this report we differentiate between these two mechanistic possibilities by characterization of a model biosynthetic intermediate containing a Dhb residue generated by the SapT biosynthetic machinery.

**Fig. 2:**
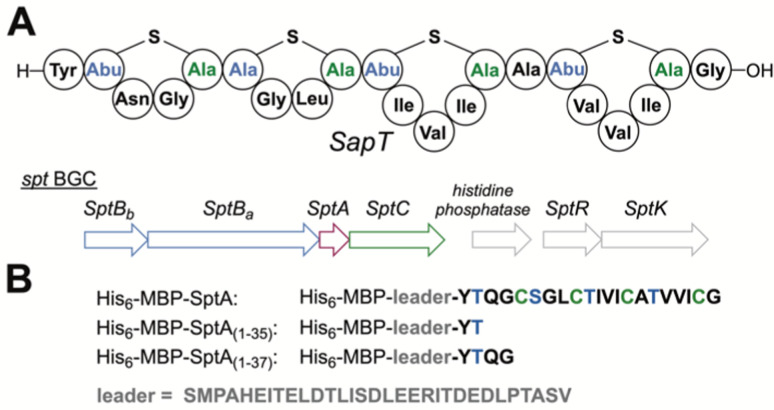
(A) Schematic structure of SapT and associated BGC. Abu, 2-aminobutyric acid. For full structures of the crosslinks, see Fig. 1. (B) Design of truncated His_6_-MBP fusion peptides used for heterologous production in this study.

The class I lanthipeptide SapT is produced from the *spt* biosynthetic gene cluster (BGC) that encodes the SptA precursor peptide, a split dehydratase consisting of SptB_a_ and SptB_b_, and the SptC cyclase (Fig. 2A).^18^ SptB_a_ is a glutamyl transferase and SptB_b_ functions as a GL that catalyzes glutamate elimination (Fig. 2A). Bioinformatic and sequence analysis previously revealed that the putative active site of SptB_b_ contains amino acid residues that are different from crystallographically characterized class I GLs that catalyze *anti*-elimination.^18,19^

The biosynthetic pathway for SapT was previously reconstituted in *Escherichia coli*,^18^ and we used this approach in this study to obtain sufficient amounts of a Dhb-containing intermediate for structural characterization. To simplify analysis, the SptA precursor peptide was truncated by site-directed mutagenesis to prepare a substrate that would generate just a single Dhb residue and that did not contain any Cys residues to avoid cyclization (Fig. 2B). Two peptides were generated, SptA_(1–37)_ and SptA_(1–35)_, both fused to the C-terminus of maltose binding protein (MBP). Co-expression of these peptides with SptB_a_ and SptB_b_, as well as glutamyl-tRNA synthase (GluRS) and tRNA^Glu^ from *Thermobispora bispora* in *E. coli* followed by isolation by metal affinity chromatography and proteolytic removal of the His_6_-MBP tag revealed dehydration of SptA_(1–37)_ to form mSptA_(1–37)_ (for modified SptA_(1–37)_) as the major product with unreacted SptA_(1–37)_ as a minor product (Fig. 3 bottom spectrum, and Table S1, ESI†). Glutamylation but not glutamate elimination was observed for SptA_(1–35)_ that contains a C-terminal Thr. The lack of elimination activity may either be the result of decreased affinity of SptB_b_ for the glutamylated peptide when situated at the C-terminus, or it may be the result of the decreased acidity of the α-C–H bond in a C-terminal Thr residue that is adjacent to a carboxylate rather than an amide (Fig. 3, Table S1, ESI†).^28^ GLs have been shown to be highly tolerant with respect to the leaving group,^29^ and hence the increased p*K*_a_ may be the most likely explanation. Regardless, we focused only on mSptA_(1–37)_ for the remainder of the study.

**Fig. 3:**
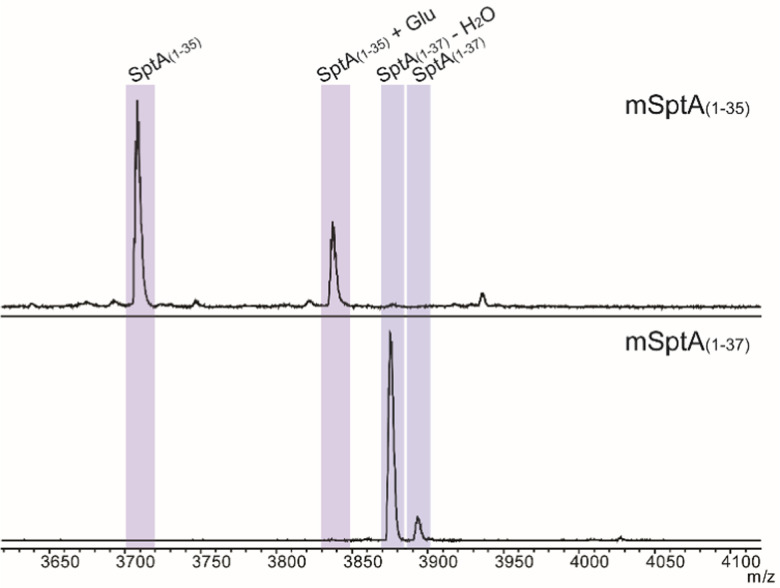
Matrix-assisted laser desorption ionization time-of-flight (MALDI-TOF) mass spectrometry analysis of truncated SptA peptides coexpressed with SptB_a_, SptB_b_, and tRNA^Glu^ and GluRS from *T. bispora*.

mSptA_(1–37)_ was digested with trypsin to remove the N-terminal portion of the peptide. The C-terminal digestion fragment (mSptA_(1–37)trypsin_) was characterized by liquid chromatography (LC) interfaced with mass spectrometry (MS). High-resolution and tandem MS confirmed the dehydration state and localized the Dhb residue to position 35 of mSptA_(1–37)_ (Fig. 4A and B and Table S2, ESI†) as expected since the other Ser/Thr residues are part of the SptA leader peptide and are not modified during the biosynthesis of SapT (Fig. 2B).

**Fig. 4:**
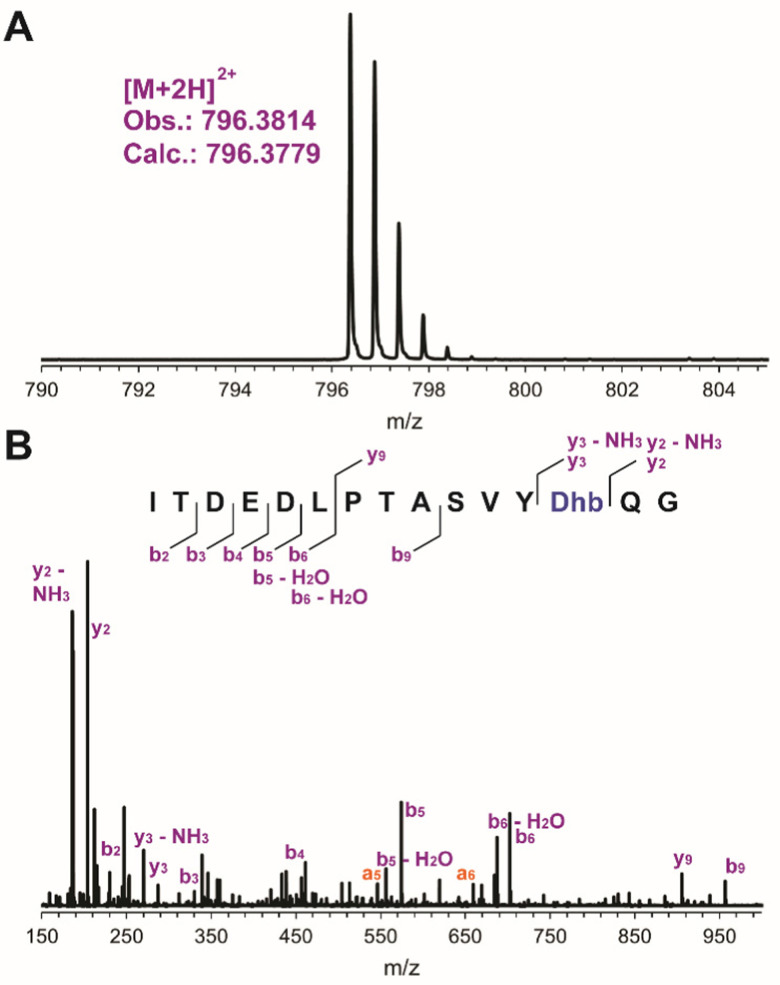
LC-MS analysis of the C-terminal trypsin digestion fragment from mSptA_(1–37)_ containing a single Dhb residue. (A) High-resolution electrospray ionization MS (ESI-MS) analysis. (B) Tandem ESI-MS analysis, observed a ions are shown in orange but not labeled on the peptide.

Next, mSptA_(1–37)trypsin_ was produced on a larger scale for characterization of the alkene geometry by nuclear magnetic resonance (NMR) spectroscopy. One dimensional ^1^H NMR spectra and two-dimensional homonuclear ^1^H-^1^H TOCSY (total correlation spectroscopy) and NOESY (nuclear Overhauser effect spectroscopy) data were acquired for chemical shift assignment of all resonances (Table S3 and Fig. S1–S4, ESI†). The ^1^H NMR spectrum of mSptA_(1–37)trypsin_ in 70% acetonitrile-d_3_/30% H_2_O revealed distinct resonance frequencies for the Dhb residue (Fig. S1 and Table S3, ESI†). The most downfield singlet at 8.90 ppm was identified as the Dhb amide proton, and a distinct quartet was observed at 5.75 ppm for the vinylic proton of the Dhb residue. Only a single set of Dhb-derived signals was detected, consistent with the MS-MS data suggesting that only Thr35 was dehydrated in mSptA_(1–37)_. The vinylic proton is coupled to the allylic methyl group of the Dhb residue at 1.85 ppm that is present as the expected doublet. An NOE signal was observed between the vinylic proton and methyl protons of the Dhb residue, as well as a distinct NOE signal between the vinylic proton of the Dhb residue and the amide proton of the same residue (Fig. 5 and Fig. S4, ESI†), revealing the spatial vicinity of the vinylic and the amide protons of the Dhb residue. An NOE signal was not observed between the methyl group and amide proton of the Dhb residue (Fig. 5 and Fig. S4, ESI†). The observed NOEs differ significantly from what is observed for (*Z*)-Dhb residues in previously characterized lanthipeptides^30,31^ (Fig. S5 and S6, ESI†) and linaridins.^32,33^ Although we cannot rule out that during purification minor products may have been removed that could contain (*Z*)-Dhb, these results clearly demonstrate that the predominant product of mSptA_(1–37)trypsin_ contains an (*E*)-Dhb and not a (*Z*)-Dhb residue. To our knowledge, this represents the first example of detection of a peptide containing an (*E*)-Dhb residue with ribosomal origin. Cypemycin had been proposed to contain an (*E*)-Dhb, but that proposal was recently shown to be incorrect.^32^ Therefore, the GL SptB_b_ in SapT biosynthesis catalyzes net *syn*-elimination of glutamylated Thr residues to form (*E*)-Dhb residues. Subsequent *anti*-addition of l-Cys across the *Si* face of the (*E*)-Dhb residue by SptC then forms d-*allo*-l-MeLan macrocycles found in SapT. Whether the formation involves a concerted *syn*-elimination reaction or potentially an E1_cb_ mechanism is currently not known. It is intriguing, however, that neither sequence alignments nor a homology model of SptB_b_ identified a residue that may stabilize the enolate intermediate that would be formed in an E1_cb_ mechanism.^19^ We cannot rule out however that amide backbone hydrogens might stabilize the build-up of negative charge on the oxygen of the enolate through hydrogen bonding interactions as often seen in proteases.^34^

**Fig. 5:**
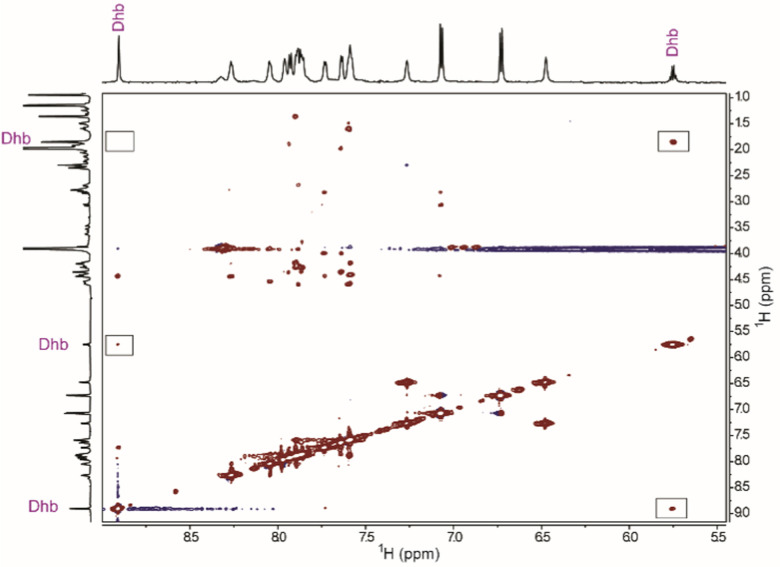
Characteristic region of the ^1^H–^1^H NOESY spectrum of mSptA_(1–37)trypsin_. Boxes are used to highlight presence and absence of key NOE signals for determining the Dhb geometry.

The characterization of a model biosynthetic intermediate containing an (*E*)-Dhb generated by SptB_b_ provides a direct link between the stereochemistry of the Dhb intermediate and bioinformatic signatures that are observed for *syn*-GLs that differ from *anti*-GLs.^18,19^ For the gene clusters that have been investigated thus far that contain a *syn*-GL (*spt* and *coi*), it is intriguing to note that the products contain d-*allo*-l-MeLan cross-links, but not (*E*)-Dhb residues.^18,19^ Whether this will hold true for other lanthipeptides containing d-*allo*-l-MeLan cross-links with *syn*-GLs in the corresponding BGCs, which constitute nearly one-fifth of all class I lanthipeptide BGCs, remains to be determined.

This study was designed by R. S and W. A. V. Experiments were performed by R. S. L. Z. assisted in NMR experiment setup. All authors contributed to data analysis and interpretation. R. S. and W. A. V. wrote the paper. All authors have given approval to the final version of the manuscript.

This work was supported by the National Institutes of Health (GM 058822). The authors thank Dr M. A. Simon for assistance with LC-MS and Prof. S. Nair and Dr Z.-F. Pei for sharing the data shown in Fig. S6 (ESI†). A Bruker MALDI TOF/TOF mass spectrometer was purchased with a grant from the National Institutes of Health (S10 RR027109 A). This study is subject to HHMI's Open Access to Publications policy. HHMI laboratory heads have previously granted a nonexclusive CC BY 4.0 license to the public and a sublicensable license to HHMI in their research articles. Pursuant to those licenses, the author-accepted manuscript of this article can be made freely available under a CC BY 4.0 license immediately upon publication.

## Conflicts of interest

There are no conflicts to declare.

## Supplementary Material

CC-059-D2CC06345J-s001
