# Supplementary material for: syn-Elimination of glutamylated threonine in lanthipeptide biosynthesis
Source: Chem Commun (Camb). 2022 Dec 14;59(9):1165–8. doi: 10.1039/d2cc06345j (PMC9890492; doi:10.1039/d2cc06345j)
Supplement: CC-059-D2CC06345J-s001 [file CC-059-D2CC06345J-s001.pdf]

Electronic Supplementary Material (ESI) for Chemical Communications.
This journal is © The Royal Society of Chemistry 2023

Electronic Supplementary Information

syn-Elimination of Glutamylated Threonine in Lanthipeptide Biosynthesis

Raymond Sarksian[a], Lingyang Zhu[b], and Wilfred A. van der Donk[a,c*]

[a]Department of Chemistry and Howard Hughes Medical Institute, University of Illinois at Urbana-Champaign, Urbana, IL, 61822, USA
[b]School of Chemical Sciences NMR Laboratory, University of Illinois at Urbana-Champaign, Urbana, IL, 61822, USA
[c]Carl R. Woese Institute for Genomic Biology, University of Illinois at Urbana-Champaign, Urbana, IL, 61822, USA
*corresponding author: vddonk@illinois.edu; 217 244 5360

Table of Contents

Site-directed Mutagenesis (SDM) to generate SptA$_{(1-35)}$ and SptA$_{(1-37)}$ 2
Small scale heterologous production and isolation of peptides 2
Large scale heterologous production and isolation of mSptA$_{(1-37)}$.. 3
HPLC purification of mSptA$_{(1-37)}$... 3
Trypsin digestion of mSptA$_{(1-37)}$ and HPLC purification 3
Trypsin digestion of mSptA$_{(1-37)}$ and analysis by LC-MS and LC-MS/MS................................. 3
NMR experiments and analysis.. 4
References.. 10

All primary data associated with this study have been deposited in a publically accessible database: https://data.mendeley.com/datasets/b3hc35g95n/1

Site-directed Mutagenesis (SDM) to generate $SptA_{(1\text{-}35)}$ and $SptA_{(1\text{-}37)}$

SDM was performed using pRSFDuet-1 His_6-MBP-SptA_$SptB_b$ as the template to generate pRSFDuet-1 His_6-MBP-$SptA_{(1\text{-}35)}$_$SptB_b$ and pRSFDuet-1 His_6-MBP-$SptA_{(1\text{-}37)}$_$SptB_b$. A previous report details the construction of the original pRSFDuet-1 His_6-MBP-SptA_$SptB_b$ plasmid.[1] SDM was performed according to a previously described protocol.[2] Primers used in this study are listed in the Table.

Primers used in this study.

Primer	Sequence (5' -> 3')
$SptA_{(1\text{-}35)}$_FP	CGTGTACACCtaaGGATGCAGCG
$SptA_{(1\text{-}35)}$_RP	GATGCCGTCGGCAGGTCC
$SptA_{(1\text{-}37)}$_FP	CACCCAGGGAtaaAGCGGCCTGTG
$SptA_{(1\text{-}37)}$_RP	TACACGGATGCCGTCGGC

Small scale heterologous production and isolation of peptides

Heterologous production (general protocol)

Chemically competent *E. coli* DH10β cells were co-transformed with pTRC33 GluRS_$tRNA^{Glu}$, pCDFDuet-1 $SptB_a$_SptC, and either pRSFDuet-1 His_6-MBP-$SptA_{(1\text{-}37)}$_$SptB_b$ or pRSFDuet-1 His6-MBP-$SptA_{(1\text{-}35)}$, and plated onto a Lysogen Broth (LB) agar plate containing 17 µg/mL kanamycin, 17 µg/mL spectinomycin, and 8 µg/mL chloramphenicol. The plate was left to incubate at 37 ^{o}C overnight and a single colony was picked the next day and grown overnight in 5 mL of Terrific Broth (TB) media containing 0.4% glycerol and the appropriate antibiotics. After overnight growth, 1 mL of the culture was used to inoculate 100 mL of fresh TB media containing 0.4% glycerol and appropriate antibiotics. The 100 mL culture was initially incubated at 37 ^{o}C while shaking at 160 rpm with monitoring OD_{600}. Once OD_{600} reached 0.8, the temperature of the incubator was lowered to 18 ^{o}C and 50 µL of 1 M IPTG (isopropyl β-D-1-thiogalactopyranoside) was added. The culture was then grown for an additional 18 h at 18 ºC. The cells were then harvested at 5000 xg for 20 min at 4 ^{o}C. Cell pellet was stored at -80 ºC until further use.

Isolation and Analysis of Peptides

The cell pellet was resuspended in 5 mL of start buffer (20 mM Tris, 500 mM NaCl, pH 7.5) and lysed by sonication. Sample was then centrifuged at 25,000 xg for 45 min at 4 ^{o}C. The supernatant was isolated and loaded onto a pre-equilibrated Ni-NTA column containing 0.5 mL of HisPur Ni-NTA resin. The column was then washed with 5 mL of wash buffer (20 mM Tris, 500 mM NaCl, 30 mM imidazole, pH 7.5) and His_6-MBP tagged peptide was eluted with 5 mL of elution buffer (20 mM Tris, 500 mM NaCl, 500 mM imidazole, pH 7.5). The elution fractions were concentrated using an Amicon Ultra-15 filter (3 kDa MW CO) by centrifugation at 4,500 xg. The buffer was then exchanged to storage buffer (20 mM Tris, 500 mM KCl, pH 7.5) by repeating the centrifugation step.

The His_6-MBP tag was removed by treating the sample in storage buffer with His_6-TEV protease (10:1 sample:protease) overnight. Samples were then desalted using C18 Ziptips (Agilent) and analyzed by matrix assisted laser desorption ionization-time of flight (MALDI-TOF) mass spectrometry by co-spotting the sample with super DHB onto a MALDI plate. MALDI-TOF MS data was acquired on a Bruker UltrafleXtreme MALDI TOF/TOF mass spectrometer.

Table S1. Observed and calculated $[M+H]^+$ m/z ratios for main **Figure 3**.

Species	Observed m/z	Calculated m/z
$SptA_{(1-35)}$	3706.1	3704.8
$SptA_{(1-35)}$ + Glu	3835.2	3833.9
$SptA_{(1-37)}$	3891.3	3889.9
$SptA_{(1-37)} - H_2O$	3873.4	3871.8

Large scale heterologous production and isolation of $mSptA_{(1-37)}$

Large scale heterologous production of MBP-$mSptA_{(1-37)}$ was performed on a 15 L scale by scaling up the small scale procedure described above using 15 individual 4 L flasks containing 1 L media for each flask.

HPLC purification of $mSptA_{(1-37)}$

$mSptA_{(1-37)}$ produced on a large scale was purified by preparative high-performance liquid chromatography (HPLC) on an Agilent Infinity 1260 system. HPLC purification of $mSptA_{(1-37)}$ was performed using a Macher Nagel C18 Htec column (10 µm particle size, 100 A pore size, 250 x 21 mm) using buffer A (0.1% TFA in H_2O) and buffer B (0.1% TFA in MeCN). Initial wash was performed using 98% buffer A and 2% buffer B for 6 min. Buffer B was then linearly increased from 2% to 100% over 46 min. $mSptA_{(1-37)}$ eluted at 50:50 A:B with a retention time of 26.1 min. Elution fractions containing the desired peptide were combined and lyophilized to yield 2 mg of $mSptA_{(1-37)}$.

Trypsin digestion of $mSptA_{(1-37)}$ and HPLC purification

$mSptA_{(1-37)}$ that was purified by HPLC was digested with trypsin (Promega, sequencing grade) using 1 mg of peptide and 20 µg of trypsin in 5 mL of buffer containing 2 mM Tris, 50 mM KCl, 1% glycerol, pH 7.5. The sample was then subjected to HPLC purification according to the conditions described above. The C-terminal fragment of $mSptA_{(1-37)}$ (ITDEDLPTASVYDhbQG) eluted at 60:40 A:B with a retention time of 21.2 – 21.6 min. The desired processed peptide was separated from unprocessed peptide (ITDEDLPTASVYTQG) that eluted earlier with a retention time of 21.0 – 21.2 min. We did not observe any other peaks with masses corresponding to dehydrated peptides, but cannot rule out that we removed very minor amounts of other such peptides.

The digestion and purification was repeated using an additional 1 mg of $mSptA_{(1-37)}$. Elution fractions containing the desired peptide were combined and lyophilized to yield 400 of µg $mSptA_{(1-37)trypsin}$ for NMR studies.

Trypsin digestion of $mSptA_{(1-37)}$ and analysis by LC-MS and LC-MS/MS

$mSptA_{(1-37)}$ (50 µg) was digested with 1 µg of trypsin (Promega, sequencing grade) in 50 mM Tris buffer, pH 7.5 at 37 °C for 3 h. After 3 h, the sample was desalted using C18 Ziptips and eluted into a 10 µL solution of 4:1 MeCN:H_2O containing 0.1% formic acid. The digested sample was loaded onto an AdvanceBio Peptide Plus (2.7 µm particle size, 150 x 2.1 mm) column and subjected to LC-MS and LC-MS/MS according to a previously reported procedure for mSptA.[1]

Table S2. Observed and calculated $[M+H]^+$ m/z for fragments of the singly dehydrated peptide $mSptA_{(1\text{-}37)trypsin}$ identified in main **Figure 4**.

Ion	Observed m/z	Calculated m/z	Error (ppm)
y_2-NH_3	187.0719	187.0713	3.207
y_2	204.0985	204.0979	2.940
b_2	215.1388	215.1390	0.930
y_3-NH_3	270.1092	270.1084	2.962
y_3	287.1356	287.1350	2.090
b_3	330.1673	330.1660	3.937
b_4	459.2059	459.2086	5.880
a_5	546.2411	546.2406	0.915
b_5-H_2O[a]	556.2257	556.2249	1.438
b_5	574.2368	574.2355	2.264
a_6	659.3254	659.3246	1.213
b_6-H_2O[a]	669.3102	669.3090	1.793
b_6	687.3213	687.3196	2.473
y_9	905.4370	905.4363	0.773
b_9	956.4576	956.4571	0.523

[a] These ions could suggest that Thr24 might be dehydrated but we do not favor this interpretation and instead suggest that these arrive from loss of H_2O from b_5 an b_6 ions, which are observed. If these dehydrated ions came from a singly dehydrated precursor in which Thr24 were dehydrated, we would expect to see ions with non-dehydrated Thr35, which were not observed.

NMR experiments and analysis

Peptide (400 µg) was dissolved in 70% acetonitrile-d_3/30% H_2O and data were acquired on an Agilent VNMRS 750 MHz spectrometer with a 5-mm triple-resonance HCN probe at 25 °C. Chemical shifts were referenced to residual MeCN solvent peak at 1.97 ppm. **Table S3** below contains all peak assignments for $mSptA_{(1\text{-}37)trypsin}$.

Table S3. Chemical shift assignments for $mSptA_{(1\text{-}37)trypsin}$. Values listed are in units of ppm.

Number	AA	NH	αH	βH	γH	δH	others
1	I		3.89	1.93	1.48, 1.18	0.94, 0.88	
2	T	8.29	4.436	3.89	1.15		
3	D	8.25	4.53	2.78			
4	E	8.03	4.236	2.05, 1.84	2.35		
5	D	7.87	4.59	2.79, 2.68			
6	L	7.587	4.55	1.60, 1.58	1.48	0.89, 0.87	
7	P		4.40	2.22, 1.99	1.92	3.75, 3.63	
8	T	7.585	4.18	4.43	1.15		
9	A	7.89	4.26	1.365			
10	S	7.847	4.345	3.81, 3.75			
11	V	7.62	4.00	1.98	0.74		

12	Y	7.72	4.430	3.06, 2.82	ArH (2, 6): 7.07<br>ArH (3, 5): 6.73		
13	*E*-Dhb	8.904		5.75	1.853		
14	Q	7.92	4.35	2.13, 1.91	2.30	NH2:7.26,6.47	
15	G	7.96	3.80				

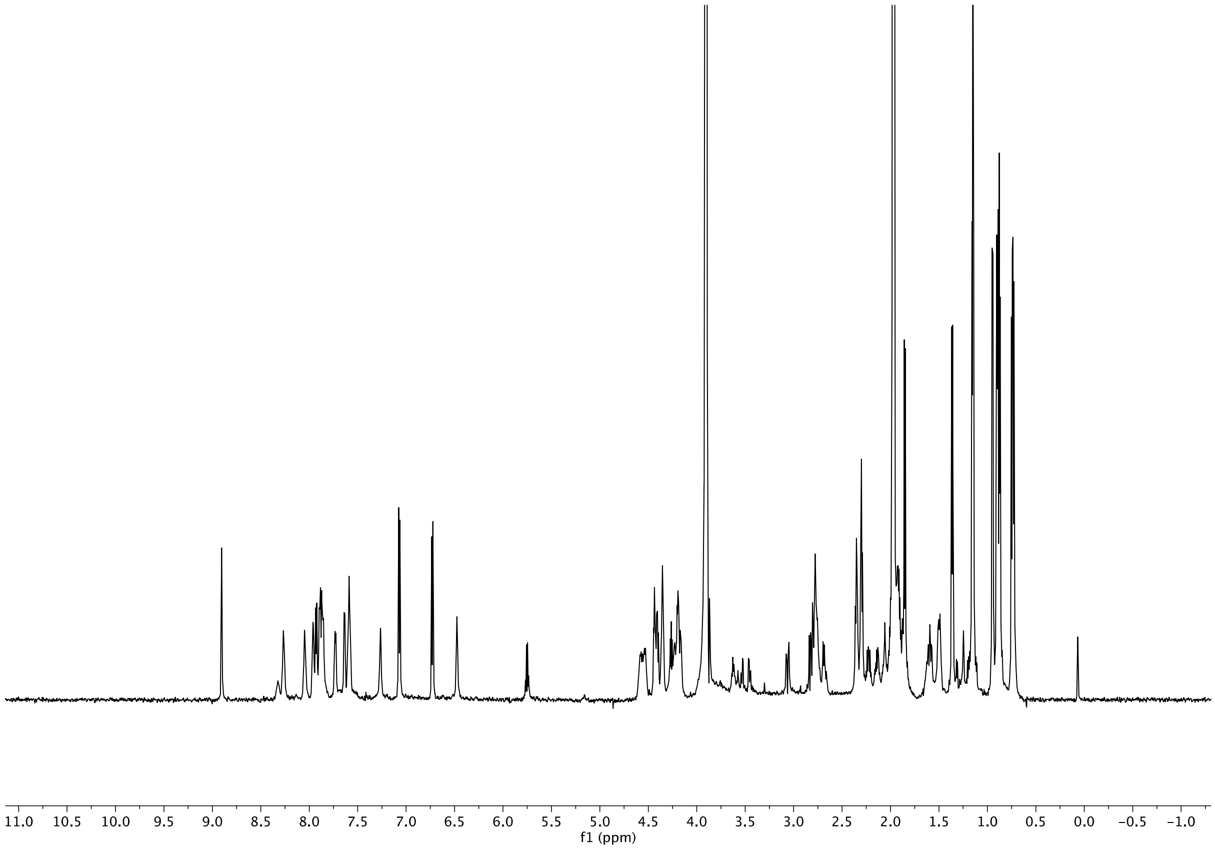

Figure S1. Water suppressed ^{1}H NMR spectrum of $mSptA_{(1\text{-}37)trypsin}$.

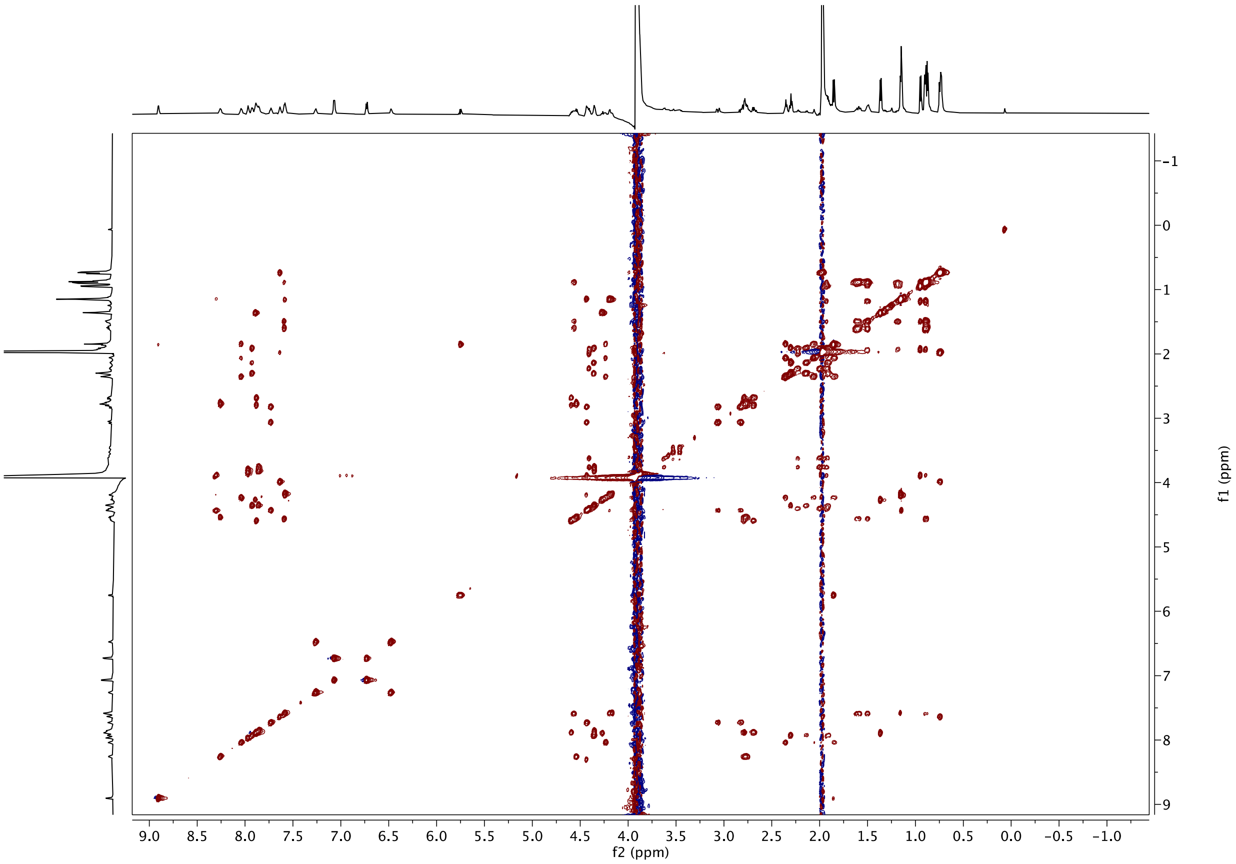

Figure S2. Water suppressed ^{1}H-^{1}H TOCSY spectrum of mSptA$_{(1-37)trypsin}$.

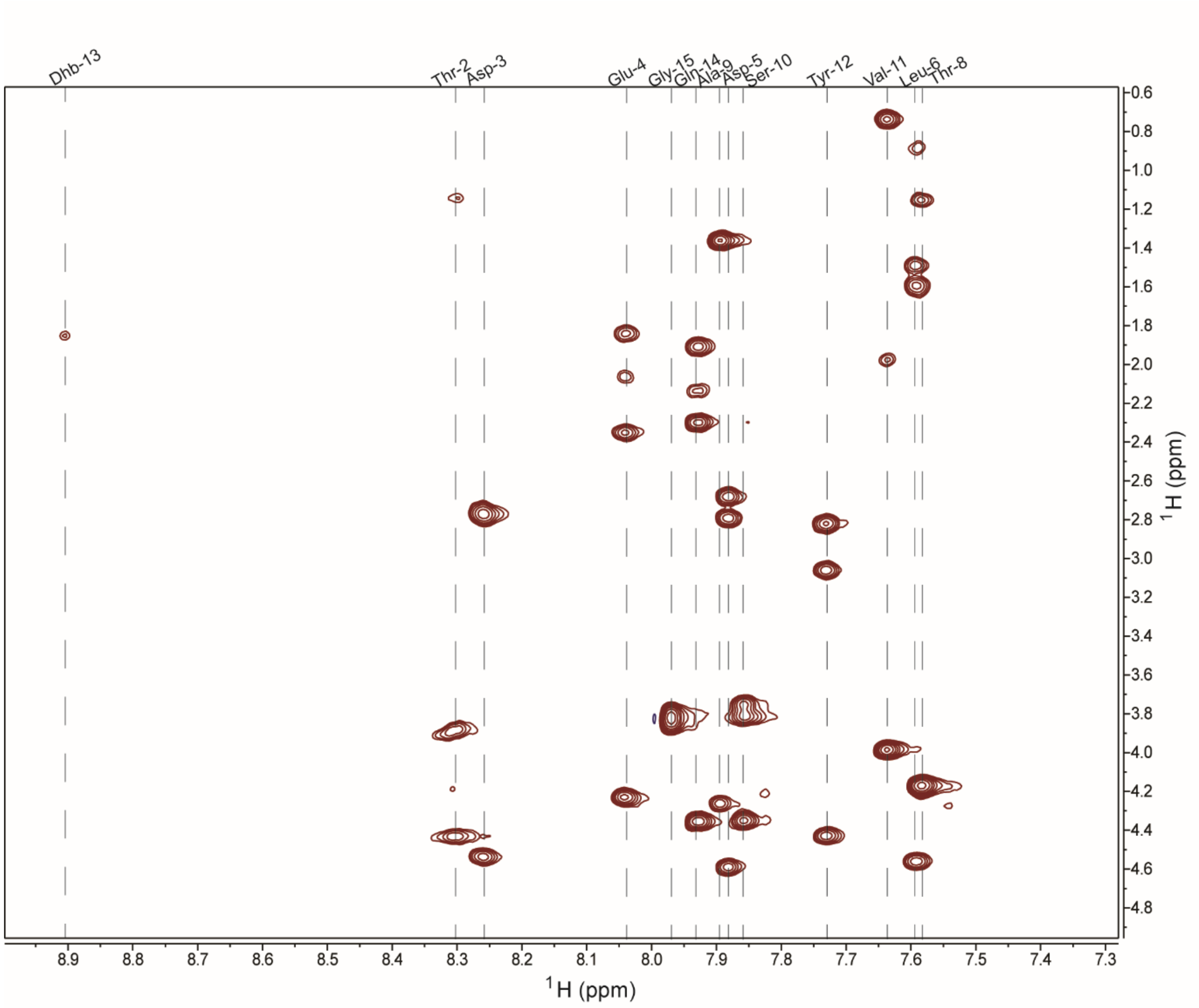

Figure S3. Water suppressed annotated ^{1}H-^{1}H TOCSY spectrum of mSptA$_{(1\text{-}37)trypsin}$ zoomed in to highlight amide proton and aliphatic proton regions.

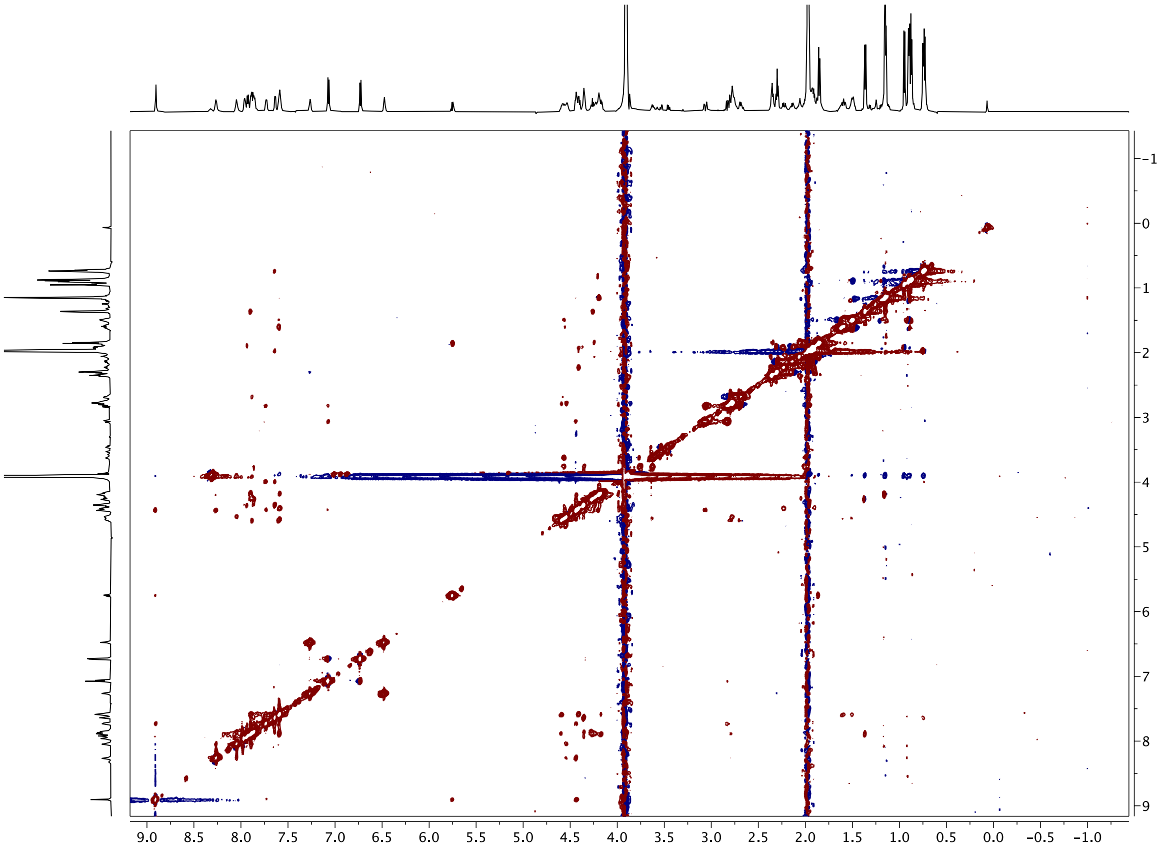

Figure S4. Water suppressed ^{1}H-^{1}H NOESY spectrum of $mSptA_{(1\text{-}37)trypsin}$.

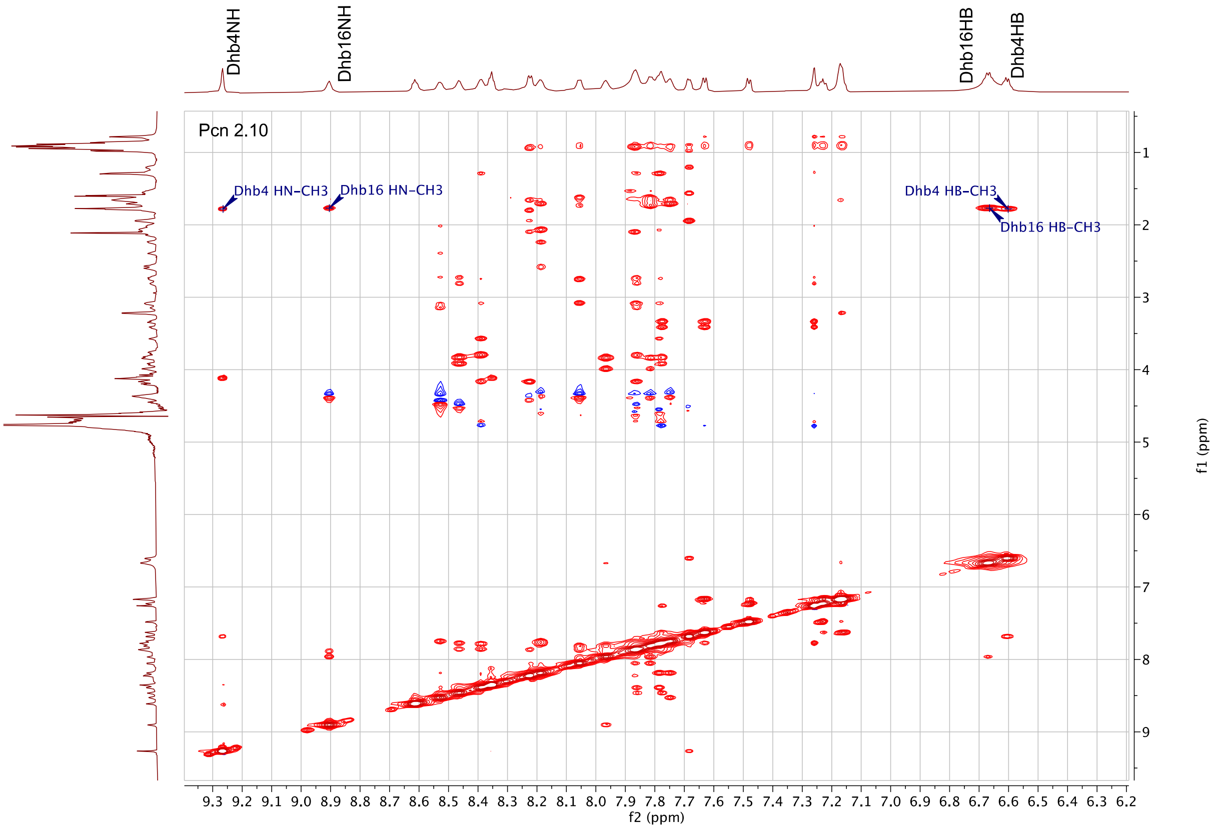

Figure S5. ^{1}H-^{1}H NOESY spectrum of previously characterized prochlorosin 2.10 that contains two (*Z*)-Dhb residues.[3] NOEs are observed between the methyl protons of Dhb residues and amide protons of the same Dhb residue. These NOEs are absent for mSptA$_{(1\text{-}37)\text{trypsin}}$ (**Figure 5 and S4**).

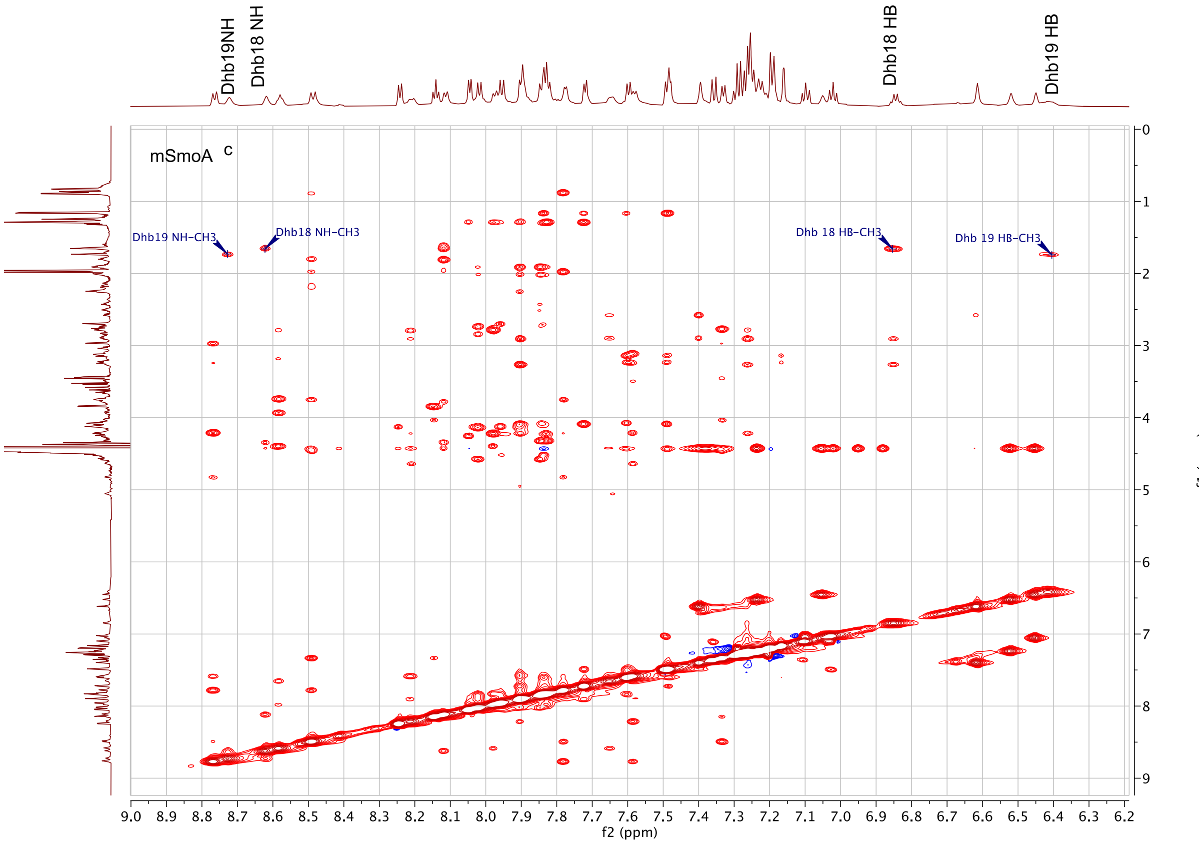

Figure S6. ^{1}H-^{1}H NOESY spectrum of mSmoA^{C} previously characterized that contains two (*Z*)-Dhb residues.[4] NOEs are observed between the methyl protons of Dhb residues and amide protons of the same Dhb residue. These NOEs are absent for mSptA$_{(1-37)trypsin}$ (**Figure 5 and S4**).

References

1. R. Sarksian, J. D. Hegemann, M. A. Simon, J. Z. Acedo and W. A. van der Donk, *J. Am. Chem. Soc.*, 2022, **144**, 6373-6382.
2. R. Sarksian and W. A. van der Donk, *ACS Chem. Biol.*, 2022, **17**, 2551-2558.
3. S. C. Bobeica, L. Zhu, J. Z. Acedo, W. Tang and W. A. van der Donk, *Chem. Sci.*, 2020, **11**, 12854-12870.
4. Z.-F. Pei, L. Zhu, R. Sarksian, W. A. van der Donk and S. K. Nair, *J. Am. Chem. Soc.*, 2022, **144**, 17549-17557.
